# Association between composite lifestyle factors and cardiometabolic multimorbidity in Chongqing, China: A cross-sectional exploratory study in people over 45 years and older

**DOI:** 10.3389/fpubh.2023.1118628

**Published:** 2023-02-01

**Authors:** Yuanjie Zheng, Zhongqing Zhou, Tingting Wu, Kailuo Zhong, Hailing Hu, Hengrui Zhang, Rong Sun, Weiwei Liu

**Affiliations:** ^1^Research Center for Medicine and Social Development, Chongqing Medical University, Chongqing, China; ^2^Research Center for Public Health Security, Chongqing Medical University, Chongqing, China; ^3^Public Health Department, Chongqing Medical University, Chongqing, China; ^4^Department of Food and Nutrition, College of Medical and Life Sciences, Silla University, Busan, South Korea; ^5^Chongqing College of Traditional Chinese Medicine, Chongqing, China; ^6^Department of Physical Examination, The First Affiliated Hospital of Chongqing Medical University, Chongqing, China

**Keywords:** multimorbidity, cardiometabolic diseases, lifestyle factors, dietary factors, dietary behavior, taste preference

## Abstract

**Introduction:**

Modifiable lifestyle factors are considered key to the control of cardiometabolic diseases. This study aimed to explore the association between multiple lifestyle factors and cardiometabolic multimorbidity.

**Methods:**

A total of 14,968 participants were included in this cross-sectional exploratory study (mean age 54.33 years, range 45–91; 49.6% male). Pearson's Chi-square test, logistic regression, and latent class analysis were employed.

**Results:**

We found that men with 4–5 high-risk lifestyle factors had a 2.54-fold higher risk (95% CI: 1.60–4.04) of developing multimorbidity compared to males with zero high-risk lifestyle factors. In an analysis of dietary behavior, we found that in women compared to men, over-eating (OR = 1.94, *P* < 0.001) and intra-meal water drinking (OR = 2.15, *P* < 0.001) were more likely to contribute to the development of cardiometabolic multimorbidity. In an analysis of taste preferences, men may be more sensitive to the effect of taste preferences and cardiometabolic multimorbidity risk, particularly for smoky (OR = 1.71, *P* < 0.001), hot (OR = 1.62, *P* < 0.001), and spicy (OR = 1.38, *P* < 0.001) tastes. Furthermore, “smoking and physical activity” and “physical activity and alcohol consumption” were men's most common high-risk lifestyle patterns. “Physical activity and dietary intake” were women's most common high-risk lifestyle patterns. A total of four common high-risk dietary behavior patterns were found in both males and females.

**Conclusions:**

This research reveals that the likelihood of cardiometabolic multimorbidity increases as high-risk lifestyle factors accumulate. Taste preferences and unhealthy dietary behaviors were found to be associated with an increased risk of developing cardiometabolic multimorbidity and this association differed between genders. Several common lifestyle and dietary behavior patterns suggest that patients with cardiometabolic multimorbidity may achieve better health outcomes if those with certain high-risk lifestyle patterns are identified and managed.

## Introduction

As life expectancy continues to increase globally (1), multimorbidity is becoming a worldwide public health problem due to its accompanying increase in disability and mortality, decrease in quality of life, and increased disease burden in the elderly (2). The global prevalence of multimorbidity is estimated to range from 12.9% in the general population to 95.1% in people aged 65 years and older (3). Cardiometabolic multimorbidity (CMM); a typical form of multimorbidity; refers to the presence of two or more cardiometabolic diseases, such as hypertension, diabetes, cardiovascular disease, heart disease, or stroke (4). Numerous studies have shown that CMM is associated with decreased cognitive function (5), a shorter life expectancy (2), a higher economic burden of the disease (6), and an uneven allocation of medical resources (7). These factors place a significant burden on individuals, families, and healthcare systems, and the burden is particularly pronounced in low- and middle-income countries.

The prevalence of chronic multimorbidity is increasing in low- and middle-income countries that are characterized by rapid urbanization, economic transition, and the spread of Western lifestyles (8). Abebe et al. showed that the prevalence of multimorbidity in low- and middle-income countries ranged from 19.4% to 80% among people aged 40 years and older (9). A cross-sectional survey showed that the prevalence of CMM in South Africa was 10.5% (10). A longitudinal cohort study including one million Chinese adults showed that the prevalence of CMM in the general population has more than doubled over 5 years (2010–2016) (11). Moreover, due to changes in nutrition and epidemiology, the burden of cardiometabolic disease is already significant in low- and middle-income countries. The fragility of social protection and health systems is not conducive to addressing these problems (12). Hence, exploring risk factors and management strategies for CMM may have significant implications for health at the individual, clinical, and public levels in these low- and middle-income countries.

China is the most populous developing country in the world and is currently experiencing a severe chronic disease epidemic (13). According to one survey, 42.4% of Chinese residents aged 50 and above suffer from multimorbidity (14). Chongqing, located in southwest China, is the most populous inland city in China and is considered an economic center. However, due to the city's rapid economic development and fast-paced way of life, most Chongqing residents live unhealthy lifestyles characterized by poor diets and physical inactivity (15). Numerous studies have revealed a connection between lifestyle factors and cardiometabolic multimorbidity (16–19). People who are obese, physically inactive, smokers, and drinkers are more likely to develop CMM. A healthy diet can help prevent or treat cardiometabolic diseases like dyslipidemia, hypertension, and diabetes (20). It is noteworthy that few studies have investigated the relationship between CMM and dietary behaviors (such as over-eating and intra-meal water drinking), despite it being a crucial lifestyle factor. Even fewer studies have explored how the combined effect of multiple dietary behaviors affects the occurrence of CMM. Chongqing's topography is primarily mountainous, and the city's climate is typically “wet” and “hot,” which may be partly responsible for the locals having developed strong taste preferences for spicy foods. The number of chili peppers consumed annually per person in Chongqing is reported to be up to 96.5 kg (21). A study in the Sichuan basin of China discovered that eating spicy foods increased the risk of adult abdominal obesity (22). Few previous studies, however, have investigated the relationship between taste preferences and CMM.

It must be highlighted that an individual's lifestyle is a combination of different habits and behaviors. Unhealthy lifestyle habits tend to cluster together, thereby dramatically raising the likelihood of developing multimorbidity (23). Meanwhile, there is evidence that some lifestyle factors may interact to have a synergistic effect, causing more harm when combined (24). Exploring potential lifestyle patterns that underly CMM in affected patients may be necessary for developing intervention strategies to improve their health outcomes. It is uncertain whether dietary behaviors and taste preferences are linked to CMM, and it is also unclear how multiple high-risk lifestyle factors affect the development of CMM. Therefore, we conducted the present study to investigate the association between multiple lifestyle factors; along with their cumulative effects; and the occurrence of CMM in Chongqing residents, as well as to explore potential high-risk lifestyle patterns and dietary behavior patterns of CMM patients.

## Method

### Materials and participants

This cross-sectional exploratory study used data obtained from the Health Management Center of the First Affiliated Hospital of Chongqing Medical University in China. From July 2020 to January 2022, 44,516 patients underwent a health checkup at the hospital's health management center. All participants were asked to complete a self-administered questionnaire to collect information about their demographic features (age, gender), personal and family history of diseases (diagnosed by a doctor), and lifestyle factors. Participants under 45, those who had been in Chongqing for < 6 months, and those whose responses contained logical errors or had more than 10% of their data missing from any questionnaire component were all excluded. Finally, 14,968 participants aged 45 and older were retained for analysis in this cross-sectional exploratory study. The study protocol was reviewed and approved by the First Affiliated Hospital of Chongqing Medical University's ethical committee (2020426). All patients/participants voluntarily signed an informed consent form to participate in this study.

### Assessment of lifestyle factors and other covariates

Based on earlier studies (25–27), the questionnaire was designed to gather information on lifestyle factors, including dietary intake, dietary habits (including dietary behaviors and taste preferences), physical activity, smoking, and alcohol consumption. Dietary intake was assessed by questioning participants about their average daily water consumption and average daily intake of each food group in the last month. The food groups included fresh vegetables, fresh fruits, rice, and flour staples, eggs, soybeans and soybean-based products, milk and milk-based products, meat and meat-based products, and fish and aquatic products. Participants were questioned about their dietary habits in the last month, such as breakfast frequency per week, night snacking per week, intra-meal water drinking, eating excessively fast, over-eating, and eating excessively late for dinner. The following dietary taste preferences were chosen from by the participants: salty, sweet, cold, hot, spicy, greasy, and smokey. Questions about physical activity included the participants' average weekly physical activity time and its degree of intensity. For smoking, participants were questioned regarding their current smoking habits, average daily cigarette consumption, and the total number of years they had smoked. Participants were questioned about their alcohol consumption, including average daily intake and type of alcohol (posed to both former and current drinkers).

The questionnaire also collected three covariates, including demographic characteristics (age and gender) and family history. Participants aged 45–59 years were included in the middle-aged group, while those aged 60 years and older were included in the older group. Participants who reported that at least one of their parents had two or more cardiometabolic diseases were considered to have a family history of cardiometabolic multimorbidity (19).

### Definition of high-risk lifestyle factors

We defined high-risk lifestyle factors based on data available from the study and characteristics of the Chinese population to calculate the number of high-risk lifestyle factors per participant, ranging from 0 to 5.

### Dietary intake

Dietary intake was assigned a value according to the recommended information of the Chinese Dietary Guidelines (2016 version) (28). A score of 1 was assigned if the average daily intake of each food group did not meet the standards recommended by the Chinese Dietary Guidelines. Otherwise, a score of 0 was assigned. The score range was from 0 to 9, described by median and interquartile range for the analysis. The higher the dietary intake score, the more the participants' dietary intake diverged from the guidelines. If the calculated dietary intake score was in the highest quartile, the participants were categorized as having high-risk dietary intake habits.

### Dietary habits

Dietary habits in this study encompassed both dietary behaviors and taste preferences. High-risk dietary behaviors included eating breakfast < 7 days per week on average, snacking at nighttime on one or more days per week on average, intra-meal water drinking, eating excessively fast, over-eating, and eating excessively late for dinner (29–32). The number of each participant's high-risk dietary behaviors was calculated as a score ranging from 0 to 6 and described by median and interquartile range analysis. If the number of calculated dietary behaviors was in the highest quartile, the participants were classified as having high-risk dietary behavior habits. In addition, due to the uniqueness of participants' taste preferences, this study did not conduct a comprehensive assessment of taste preferences.

### Physical activity

The recommended duration of physical activity recommended by the physical activity guidelines for Chinese people (2021 version) is 150–300 min of moderate-intensity or 75–150 min of vigorous-intensity aerobic activity per week or an equivalent combination of moderate-intensity and vigorous-intensity aerobic activity (33). Participants who did not meet the recommended criteria were considered physically inactive and hence were categorized as having high-risk physical activity habits.

### Smoking

The smoking index is defined as the product of the number of cigarettes smoked per day and the number of years of smoking, which is used to represent the cumulative effect of cumulative smoking on the health risks of smokers and ex-smokers (34). If a participant's smoking index was more than or equal to 100, they were categorized as having high-risk smoking behavior.

### Alcohol consumption

Participants' responses for average daily drinking quantity and type of wine were combined to calculate their average daily alcohol consumption according to the proportions recommended by the Chinese Dietary Guidelines (2016 version) (28). Participants were categorized as high risk if the average alcohol consumption per day exceeded the guideline's recommendation (25 g for males and 15 g for females).

### Definition of cardiometabolic multimorbidity

We collected participants' disease history through the questionnaire, which included hypertension, diabetes, dyslipidemia, obesity, coronary heart disease, stroke, and osteoporosis (35). These diseases were identified from self-reported doctor diagnoses. For this study, we defined CMM as the coexistence of two or more cardiometabolic diseases (4).

### Statistical analysis

In this study, age was expressed as the mean and standard deviation; the number of diseases and high-risk lifestyle factors were expressed as the median and range; the number of dietary intake scores and high-risk dietary behaviors were expressed as the median and interquartile spacing; categorical variables were expressed as the frequency and percentage. All analyses were stratified due to variations in the distribution of adherence to lifestyle variables by gender. Pearson's Chi-square test for categorical variables was used to analyze variance to determine the impact of various lifestyle factors on CMM. Binary logistic regression analysis was used to determine the association between lifestyle factors (individual and cumulative) and CMM. Tolerance and the variance inflation factor (VIF) were used to test for multicollinearity among independent variables. In the logistic regression model, the ratio (OR) and associated 95% confidence interval (95% CI) were determined for each component. With two-sided tests and a significance threshold of 0.05, all of the aforementioned statistical analyses were carried out using SPSS version 25.0 (SPSS, Inc., Chicago, IL, USA).

Latent class analysis (LCA) was used to further explore the potential high-risk dietary behavior and lifestyle patterns in CMM patients using Mplus version 8.3 (Muthén & Muthén, Los Angeles, CA, USA). Latent class analysis, a method for person-centered classification that combines people with similar behaviors into potential classes, is crucial for determining whether subgroups of high-risk lifestyle factors exist (36). We fitted a one-class model to identify the most concise model and steadily raised the number of potential classes. The Lo-Mendell-Rubin adjusted likelihood ratio test (LMR), bootstrapped likelihood ratio test (BLRT), entropy score, and Bayesian Information Criterion (BIC) were employed to evaluate the model's fit (37).

## Results

A total of 14,968 participants were included in this study, including 7,427 males and 7,541 females. The mean age of the participants was 54.33 (SD = 7.31) years, ranging from 45 to 91 years. Table 1 shows the characteristics of the participants' diseases and lifestyle factors. A total of 4,788 participants (32.0%) had at least one disease and 1,674 participants (11.2%) had CMM. The most common cardiometabolic diseases were hypertension (16.5%), followed by dyslipidemia (15.1%), and diabetes (6.4%). For lifestyle factors, patients with 4 and 5 high-risk lifestyle factors were combined into one group because few participants were characterized by five high-risk lifestyle factors. After stratification by gender, women were found to engage in significantly lower rates of high-risk smoking (3.5%) and high-risk alcohol consumption (13.6%) than men. The associations of participants' lifestyle factors with CMM are shown in Table 2. Physical inactivity and high-risk alcohol consumption were associated with CMM in both sexes (*P* < 0.05). For high-risk smoking, however, there was no discernible difference between the sexes (*P* > 0.05). High-risk dietary intake was associated with CMM in women only (*P* < 0.001) and high-risk dietary behavior in men only (*P* = 0.014).

**Table 1:** Characteristics of disease and lifestyle of the participants (*N* = 14,968).

		**Total**	**Male (*n* = 7,427)**	**Female (*n* = 7,541)**	***P*-value**
Age^a^		54.33 ± 7.31	54.37 ± 7.37	54.29 ± 7.27	
Family history	No	11,825 (79.0%)	5,932 (79.9%)	5,893 (78.1%)	0.010
	Yes	3,143 (21.0%)	1,495 (20.1%)	1,648 (21.9%)	
Dietary intake	Good	9,919 (66.3%)	5,005 (67.4%)	4,914 (65.2%)	0.004
	Poor	5,049 (33.7%)	2,422 (32.6%)	2,627 (34.8%)	
Dietary behavior	Good	10,243 (68.4%)	4,640 (62.5%)	5,603 (74.3%)	< 0.001
	Poor	4,725 (31.6%)	2,787 (37.5%)	1,938 (25.7%)	
Physical activity	Active	2,939 (19.6%)	1,577 (21.2%)	1,362 (18.1%)	< 0.001
	Inactive	12,029 (80.4%)	5,850 (78.8%)	6,179 (81.9%)	
High-risk smoking	No	11,917 (79.6%)	4,484 (60.4%)	7,433 (98.6%)	< 0.001
	Yes	3,051 (20.4%)	2,943 (39.6%)	108 (1.4%)	
High-risk alcohol consumption	No	10,854 (72.5%)	3,873 (52.1%)	6,981 (92.6%)	< 0.001
	Yes	4,114 (27.5%)	3,554 (47.9%)	560 (7.4%)	
Hypertension	No	12,497 (83.5%)	5,853 (78.8%)	6,644 (88.1%)	< 0.001
	Yes	2,471 (16.5%)	1,574 (21.2%)	897 (11.9%)	
Diabetes	No	14,007 (93.6%)	6,780 (91.3%)	7,227 (95.8%)	< 0.001
	Yes	961 (6.4%)	647 (8.7%)	314 (4.2%)	
Coronary Heart Disease	No	14,623 (97.7%)	7,212 (97.1%)	7,411 (98.3%)	< 0.001
	Yes	345 (2.3%)	215 (2.9%)	130 (1.7%)	
Dyslipidemia	No	12,701 (84.9%)	6,079 (81.9%)	6,622 (87.8%)	< 0.001
	Yes	2,267 (15.1%)	1,348 (18.1%)	919 (12.2%)	
Obesity	No	14,560 (97.3%)	7,134 (96.1%)	7,426 (98.5%)	< 0.001
	Yes	408 (2.7%)	293 (3.9%)	115 (1.5%)	
Stroke	No	14,899 (99.5%)	7,383 (99.4%)	7,516 (99.7%)	0.018
	Yes	69 (0.5%)	44 (0.6%)	25 (0.3%)	
Osteoporosis	No	14,343 (95.8%)	7,215 (97.1%)	7,128 (94.5%)	< 0.001
	Yes	625 (4.2%)	212 (2.9%)	413 (5.5%)	
Number of high-risk lifestyle factors^b^	2 (0–5)	2 (0–5)	1 (0–5)		
0		1,036 (6.9%)	1,036 (6.9%)	317 (4.3%)	< 0.001
1		4,743 (31.7%)	4,743 (31.7%)	1,491 (20.1%)	
2		4,932 (33.0%)	4,932 (33.0%)	2,287 (30.8%)	
3		2,910 (19.4%)	2,910 (19.4%)	2,075 (27.9%)	
4-5		1,347 (9.0%)	1,347 (9.0%)	1,257 (16.9%)	
Number of cardiometabolic diseases^b^		0 (0–7)	0 (0–7)	0 (0–7)	
0		10,180 (68.0%)	4,606 (62.0%)	5,574 (73.9%)	< 0.001
1		3,114 (20.8%)	1,765 (23.8%)	1,349 (17.9%)	
2		1,165 (7.8%)	718 (9.7%)	447 (5.9%)	
3		389 (2.6%)	255 (3.4%)	134 (1.8%)	
4		89 (0.6%)	62 (0.8%)	27 (0.4%)	
5-7		31 (0.2%)	21 (0.3%)	10 (0.1%)	
Dietary intake scores^c^		7 (6-8)	7 (6-8)	7 (6-8)	
Number of high-risk dietary behaviors^c^	0 (0–2)	1 (0–2)	0 (0–2)		

^a^refers to data were presented by mean and SD.

^b^refers to data were presented by median and range.

^c^refers to data were presented by Median interquartile range.

**Table 2:** Association of participant lifestyle factors with cardiometabolic multimorbidity (*N* = 14,968).

		**Male (*****n*** = **7,427)**			**Female (*****n*** = **7,541)**		
		**Total**	**Multimorbidity/n (%)**	***P*** **-value**	**Total**	**Multimorbidity/*****n*** **(%)**	***P*** **-value**
Age group	45–59	6,011	725 (12.1%)	< 0.001	6,124	315 (5.1%)	< 0.001
	≥60	1,416	331 (23.4%)		1,417	303 (21.4%)	
Family History	No	5,932	622 (10.5%)	< 0.001	5,893	340 (5.8%)	< 0.001
	Yes	1,495	434 (29.0%)		1,648	278 (16.9%)	
**Lifestyle factors**							
Dietary intake	Good	5,005	693 (13.8%)	0.187	4,914	363 (7.4%)	< 0.001
	Poor	2,422	363 (15.0%)		2,627	255 (9.7%)	
Dietary behavior	Good	4,640	624 (13.4%)	0.014	5,603	449 (8.0%)	0.328
	Poor	2,787	432 (15.5%)		1,938	169 (8.7%)	
Physical activity	Active	1,577	196 (12.4%)	0.022	1,362	88 (6.5%)	0.010
	Inactive	5,850	860 (14.7%)		6,179	530 (8.6%)	
High-risk smoking	No	4,484	630 (14.0%)	0.608	7,433	612 (8.2%)	0.314
	Yes	2,943	426 (14.5%)		108	6 (5.6%)	
High-risk alcohol consumption	No	3,873	512 (13.2%)	0.010	6,981	585 (8.4%)	0.039
	Yes	3,554	544 (15.3%)		560	33 (5.9%)	
**Number of high-risk lifestyle factors**							
0		317	23 (7.3%)	< 0.001^a^	719	45 (6.3%)	0.004^a^
1		1,491	190 (12.7%)		3,252	243 (7.5%)	
2		2,287	330 (14.4%)		2,645	247 (9.3%)	
3		2,075	315 (15.2%)		835	76 (9.1%)	
4–5		1,257	198 (15.8%)		90	7 (7.8%)	

^a^refers to P-value for trend.

The odds ratio (OR) of CMM associated with the number of high-risk lifestyle factors is shown in Table 3. After adjusting for age group and family history, the results varied significantly between the sexes. Men with 4 to 5 high-risk lifestyle factors had a 2.54-fold higher risk (95% CI: 1.60–4.04) of developing multimorbidity compared to males without any high-risk lifestyle factors. Two or three high-risk lifestyle factors considerably increased the probability of CMM in women. The trend test (in Table 2) for the number of high-risk lifestyle factors with CMM was significant in both men (*P* < 0.001) and women (*P* = 0.004). Accumulating the number of high-risk lifestyle factors was positively and significantly associated with an increased likelihood of CMM in both genders.

**Table 3:** Logistic regression of the number of high-risk lifestyle factors and the likelihood of cardiometabolic multimorbidity.

	**Male**		**Female**	

	**OR**	**95% CI**	**OR**	**95% CI**
**Number of high-risk lifestyles (ref. 0)**				
1	1.85	(1.17–2.94)^*^	1.12	(0.79–1.58)
2	2.21	(1.41–3.47)^**^	1.63	(1.15–2.30)^*^
3	2.48	(1.58–3.90)^**^	1.80	(1.20–2.69)^*^
4–5	2.54	(1.60–4.04)^**^	1.78	(0.76–4.18)

Adjustment with age group and family history of cardiometabolic multimorbidity,^*^P < 0.01, ^**^P < 0.001.

**Figure 1:**
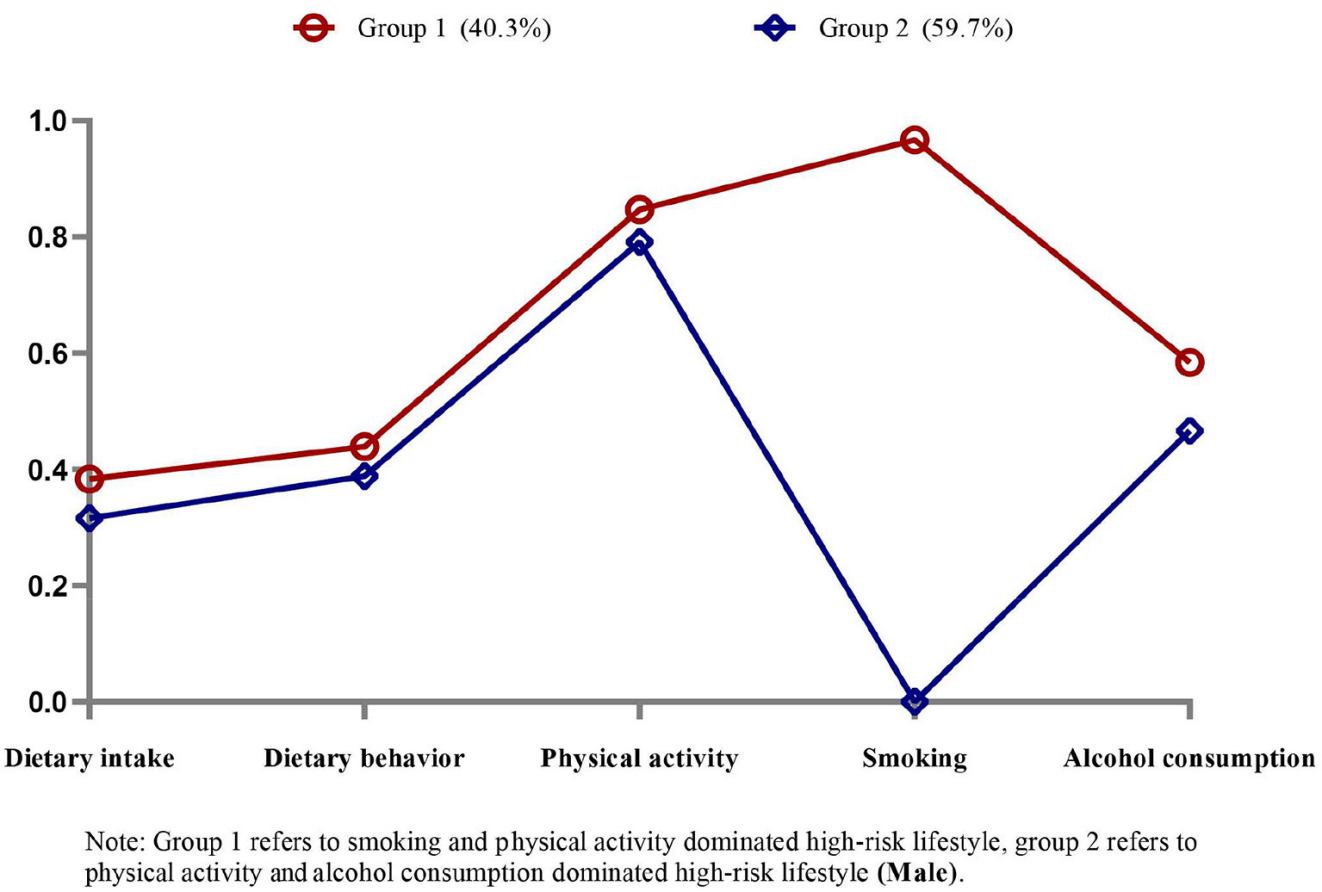
Item probabilities in the two common high-risk lifestyle patterns among male cardiometabolic multimorbidity patients.

**Figure 2:**
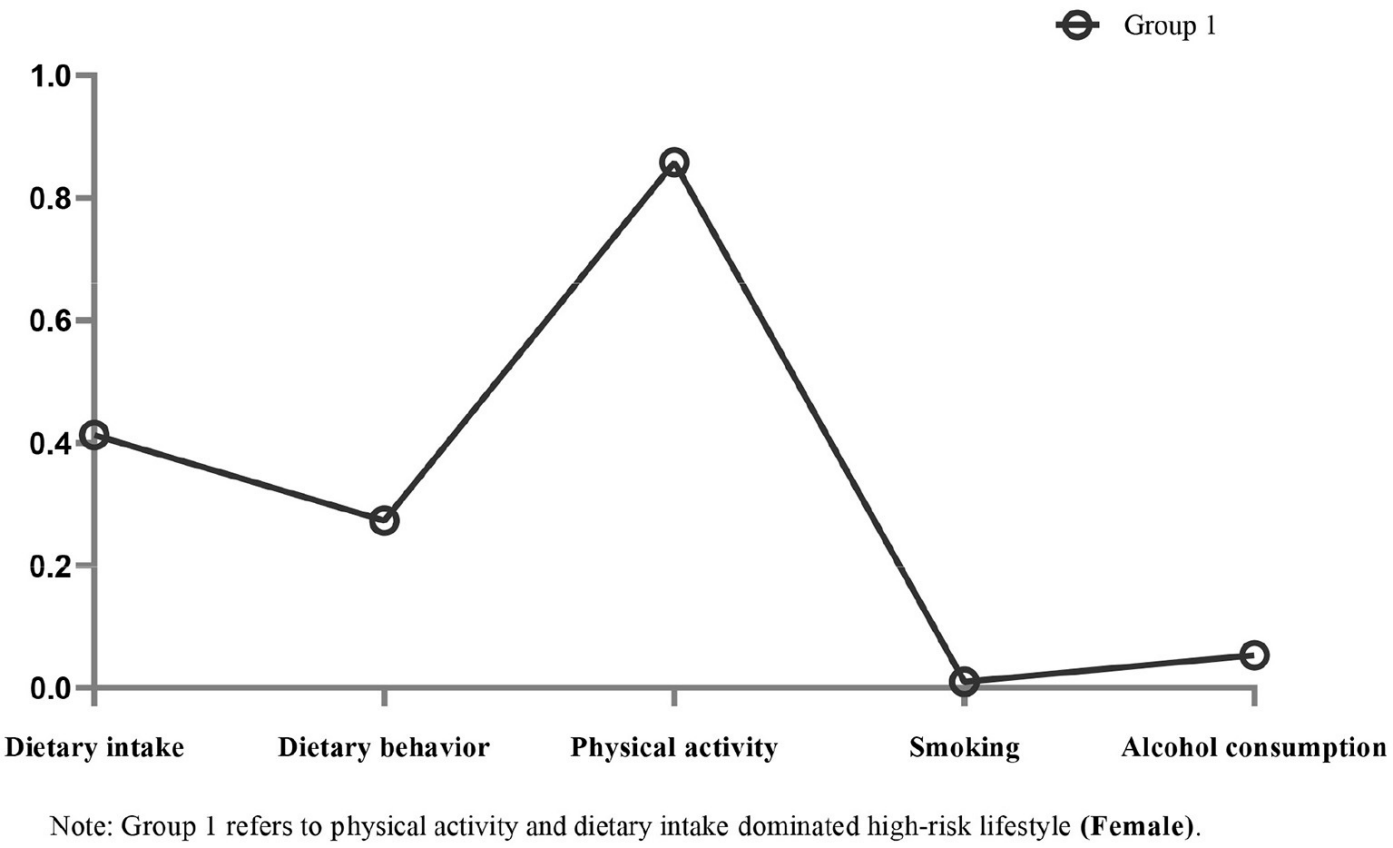
Item probabilities in the one common high-risk lifestyle pattern among female cardiometabolic multimorbidity patients.

Figure 1 shows each model's conditional probabilities for each high-risk lifestyle factor among male CMM patients. Based on the results of the LCA model's relevant statistical indicators, we found that the two-class model with the lower BIC and ABIC values, significant LMR and BLRT values, and high entropy score (0.906) was the best (Supplementary Table 1). Male patients with CMM had two common high-risk lifestyle patterns. Group 1 (*N* = 426, 40.3%) consisted of individuals classified as having a “high-risk lifestyle dominated by high-risk smoking and high-risk physical activity.” Similarly, group 2 (*N* = 630, 59.7%) was categorized as having “high-risk lifestyles dominated by high-risk physical activity and high-risk alcohol consumption.” Figure 2 shows the model's conditional probabilities for each high-risk lifestyle factor among female CMM patients. Based on the LCA model's relevant statistical indicators, we found that the one-class model was the best (Supplementary Table 2). Female patients with CMM had one common high-risk lifestyle pattern. Group 1 (*N* = 618, 100%) consisted of individuals classified as having a “high-risk lifestyle dominated by high-risk physical activity and high-risk dietary intake.” The results show that individuals in three subgroups had a high probability of having high-risk physical activity. Thus, high-risk physical activity is an important lifestyle factor that dominates the high-risk lifestyle pattern of CMM patients.

**Figure 3:**
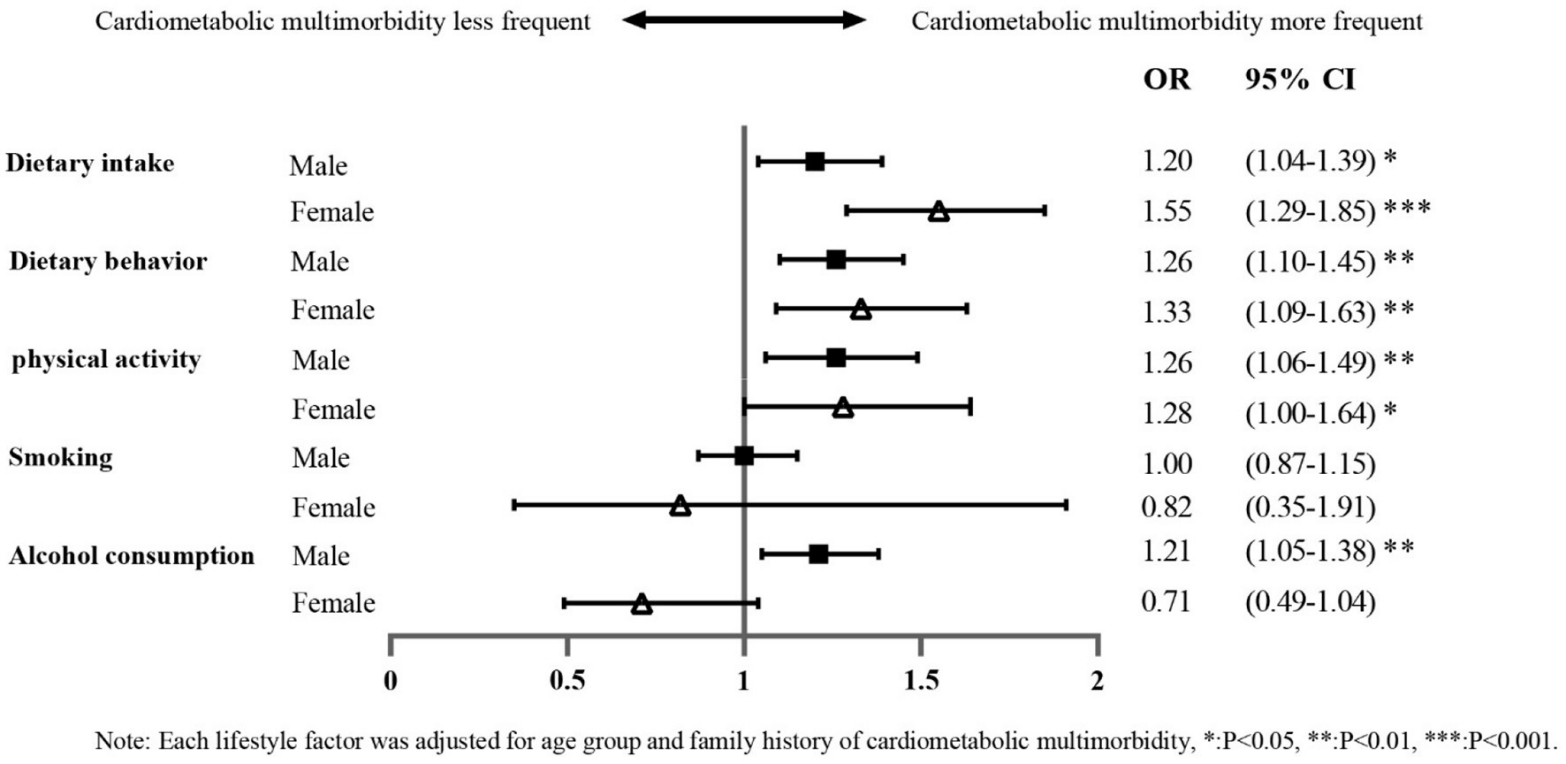
Forest plot showing the bivariate effect of each high-risk lifestyle factor on the likelihood of cardiometabolic multimorbidity.

The odds ratio (OR) of the binary effect of each lifestyle factor on the presence of CMM after adjusting for age group and family history is shown in Figure 3. The effect of lifestyle factors on CMM was found to vary by gender. Men (OR: 1.20; 95% CI: 1.04–1.39) and women (OR: 1.55; 95% CI: 1.29–1.85) with high-risk dietary intake had a higher risk of CMM than participants without high-risk dietary intake. Men with high-risk dietary behavior were 1.26-fold (95% CI: 1.10–1.45) more likely to report CMM than men without high-risk dietary behavior, corresponding to 1.33-fold (95% CI: 1.09–1.63) for women. Physical inactivity was a risk factor for CMM in both men (OR: 1.26; 95% CI: 1.06–1.49) and women (OR: 1.28; 95% CI: 1.00–1.64). The association between high-risk smoking and CMM in both sexes was not statistically significant. High-risk alcohol consumption (compared to low-risk drinking) was a significant risk factor in men only (OR: 1.21; 95% CI: 1.05–1.38).

Table 4 shows the odds ratio (OR) for the binary effect of each dietary habit (including dietary behavior and taste preference) on the presence of cardiometabolic multimorbidity, adjusted for age group and family history. The association between dietary behavior and CMM varied by gender. For women, intra-meal water drinking (OR: 2.15; 95% CI: 1.60–2.89), over-eating (OR: 1.94; 95% CI: 1.49–2.53), and eating excessively late for dinner (OR: 1.53; 95% CI: 1.16–2.03) were associated with an increased likelihood of CMM. In contrast, among men, eating excessively fast (OR: 1.64; 95% CI: 1.42–1.89) and over-eating (OR: 1.54; 95% CI: 1.27–1.86) were risk factors for the development of CMM. The association of taste preferences with CMM varied by gender. In women, taste preferences for salty (OR: 1.72; 95% CI: 1.28–2.32) and greasy (OR: 1.75; 95% CI: 1.23–2.49) foods were associated with a higher likelihood of CMM, and the negative health effects were more prominent in women than in men. However, taste preferences for spicy (OR: 1.38; 95% CI: 1.18–1.61), smoky (OR: 1.71; 95% CI: 1.31–2.24), and hot (OR: 1.62; 95% CI: 1.29–2.04) foods affected the occurrence of CMM in men only.

**Table 4:** Logistic regression of the independent effects of each dietary behavior and taste preference on cardiometabolic multimorbidity.

	**Male**		**Female**	

	**OR**	**95% CI**	**OR**	**95% CI**
**Dietary behavior**				
Breakfast irregularity (ref. No)				
Yes	0.88	(0.72–1.04)	0.81	(0.63–1.06)
Frequent night snacking (ref. No)				
Yes	1.03	(0.90–1.18)	1.17	(0.97–1.40)
Intra-meal water drinking (ref. No)				
Yes	1.14	(0.87–1.50)	2.15	(1.60–2.89)^**^
Eating excessively fast (ref. No)				
Yes	1.64	(1.42–1.89)^**^	1.18	(0.95–1.48)
Over-eating (ref. No)				
Yes	1.54	(1.27–1.86)^**^	1.94	(1.49–2.53)^**^
Eating excessively late for dinner (ref. No)				
Yes	1.18	(0.95–1.48)	1.53	(1.16–2.03)^*^
**Taste preference**				
Salty (ref. No)				
Yes	1.57	(1.30–1.91)^**^	1.72	(1.28–2.32)^**^
Hot (ref. No)				
Yes	1.62	(1.29–2.04)^**^	1.19	(0.80–1.77)
Greasy (ref. No)				
Yes	1.55	(1.55–2.30)^**^	1.75	(1.23–2.49)^*^
Smoked (ref. No)				
Yes	1.71	(1.31–2.24)^**^	1.47	(0.97–2.23)
Spicy (ref. No)				
Yes	1.38	(1.18–1.61)^**^	1.13	(0.89–1.44)

Adjustment with age group and family history of cardiometabolic multimorbidity,^*^P < 0.01,^**^P < 0.001.

**Figure 4:**
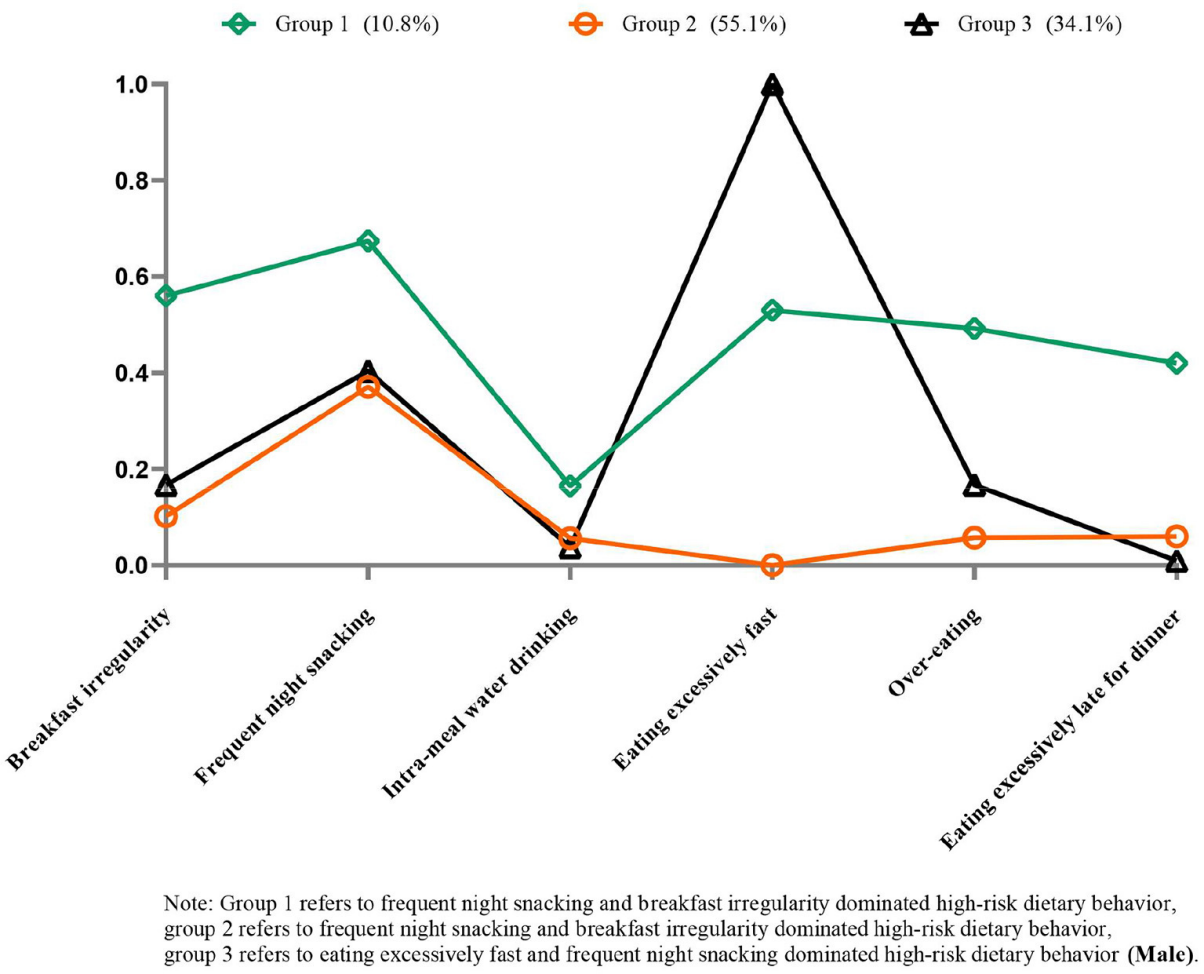
Item probabilities in the three common high-risk dietary behavior patterns among male cardiometabolic multimorbidity patients.

Figure 4 shows each model's conditional probabilities of high-risk dietary behavior among male CMM patients. Based on the results of the LCA model's relevant statistical indicators, the three-class model with lower BIC and ABIC values, significant LMR and BLRT values, and a high entropy score (0.767) was determined to be the best (Supplementary Table 3). Male patients with CMM were characterized by one of two common patterns of high-risk dietary behaviors. Group 1 (*N* = 114, 10.8%) and group 2 (*N* = 582, 55.1%) consisted of individuals categorized as having “high-risk dietary behavior dominated by frequent night snacking and breakfast irregularity.” Similarly, group 3 (*N* = 360, 34.1%) was classified as having “high-risk dietary behaviors dominated by eating excessively fast and frequent night snacking.”

**Figure 5:**
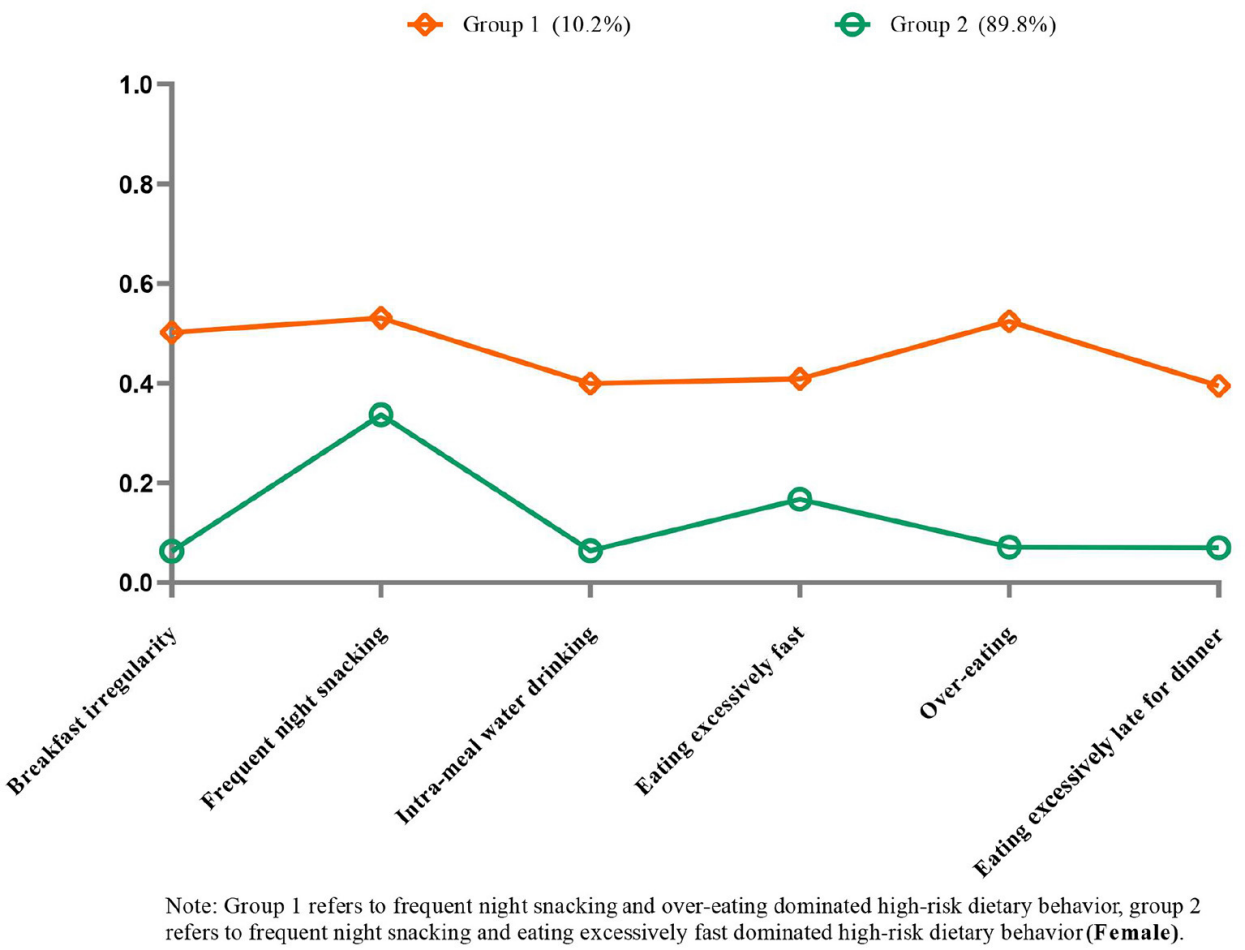
Item probabilities in the two common high-risk dietary behavior patterns among female cardiometabolic multimorbidity patients.

Figure 5 shows each model's conditional probabilities of high-risk dietary behavior among female CMM patients. Based on the results of the LCA model's relevant statistical indicators, the two-class model with lower BIC and ABIC values, significant LMR and BLRT values, and a high entropy score (0.717) was determined to be the best (Supplementary Table 4). Female patients with CMM were characterized by one of two common patterns of high-risk dietary behaviors. Group 1 (*N* = 63, 10.2%) consisted of individuals categorized as having “high-risk dietary behavior dominated by frequent night snacking and over-eating.” Similarly, group 2 (*N* = 555, 89.8%) was classified as having “high-risk dietary behaviors dominated by frequent night snacking and eating excessively fast.”

## Discussion

This study suggests that several common lifestyle factors and their combined effects are associated with the likelihood of CMM in middle-aged and older adults. Upon inspecting the lifestyle factors individually, those that may be associated with CMM include physical activity, alcohol consumption, dietary intake, and dietary behavior. The likelihood of CMM gradually increased with the accumulation of high-risk lifestyle factors when lifestyle factors were combined, starting from a threshold of one lifestyle factor in men and two in women.

Shao et al. found that participants who accumulated more unhealthy lifestyle behaviors had a higher likelihood of cardiometabolic multimorbidity, which is consistent with our findings in men (38). Similar to the findings of Fortin et al. (39), we found that two or three unhealthy lifestyle factors increased the likelihood of multimorbidity in women compared to those with none. Furthermore, our findings revealed that, while not significant on its own in men, high-risk smoking was most strongly related to the development of CMM when combined with other factors. Thus, we predicted that the total probability of CMM development might be influenced by the number of accumulated high-risk lifestyle factors and their specific combinations (38, 40). We discovered two common high-risk lifestyle patterns among male CMM patients: “smoking and physical activity” and “physical activity and alcohol consumption”. These findings support our hypothesis that certain lifestyle factors can coexist and interact to have synergistic effects (24). Meanwhile, we discovered only one common high-risk lifestyle pattern among female CMM patients: “physical activity and dietary intake”. The low rates of high-risk smoking and high-risk alcohol consumption among women may explain this result. Notably, people in three subcategories were highly likely to have engaged in high-risk physical activity, indicating that other high-risk lifestyle factors may frequently accompany these behaviors in CMM patients. This finding helps to explain why the healthcare system should shift its focus to addressing multiple behaviors for multiple diseases.

Numerous critical studies have shown that CMM is associated with inactive physical activity, smoking, and high-risk alcohol consumption in middle-aged and older adults (16–18). Our study confirmed physical inactivity to be a risk factor for CMM. However, there was a lack of association between high-risk alcohol consumption and high-risk smoking and CMM in women. We found only a low percentage of women to be characterized by high-risk alcohol consumption and high-risk smoking, regardless of cardiometabolic multimorbidity, which is consistent with other studies (41) and may explain the results. The findings could also be due to fundamental differences in the distribution of these factors between the sexes, or they could be related to historical and societal factors that impact women's smoking and alcohol-drinking habits (42). In addition, this study found the same lack of association between high-risk smoking with CMM in men, which may be related to the characteristics of the low-risk smoking population. We found a higher proportion of the remaining four high-risk lifestyle factors among men with low-risk smoking. Moreover, our data analysis showed that all four high-risk lifestyle factors, except smoking, were risk factors for CMM in men. The remaining high-risk lifestyle factors likely amplify the dangerous effect of smoking behavior in the low-risk smoking group, thereby diluting the effect of high-risk smoking on CMM.

A longitudinal study from Jiangsu, China, found that the high consumption of fruits and vegetables reduced the risk of CMM by analyzing the association between the average daily intake of multiple foods and multiple morbidities (26), which is consistent with findings obtained in the United States and Korea, among other countries (25, 35). However, this contrasts with our findings, which indicated that fruit and vegetable intake was not significantly associated with the risk of CMM (Supplementary Table 5). We surmise that this may be due to a significant fraction of participants (almost 80%) not achieving the recommended intake of fruits and vegetables, which could dampen the risk effect. It also implies that steps must be taken to modulate this behavior in the population, as the general residents of Chongqing may be at risk of consuming too few fruits and vegetables. In addition, we investigated the relationship between the high-risk intake of nine food groups and CMM. The results confirm the findings reported by Micha et al., namely, that nine food groups were significantly associated with CMM when evaluated collectively (25). Notably, the adverse health effects associated with high-risk dietary intake were higher in women than men. However, caution should be exercised in interpreting the results due to the inherent limitations of conducting dietary intake assessments (42), such as methodological differences in measuring different food intakes and the different risk intake thresholds identified.

Individual high-risk dietary behaviors; such as breakfast irregularity (43), frequent night snacking (29), over-eating (44), and eating excessively fast (30); have a large body of evidence supporting them as risk factors for cardiometabolic disease. Our findings are generally consistent with previous studies. According to a Brazilian longitudinal study (45), over-eating was associated with a higher likelihood of developing cardiometabolic diseases. We further discovered gender-specific differences in this relationship. The adverse health effects of over-eating were more prominent in women than in men, which supports the findings of a Canadian longitudinal study (46). Additionally, present knowledge about the association between dietary behavior and CMM is limited. For example, there is no data on the relationship between intra-meal water drinking and CMM. An Iranian study found that intra-meal water drinking increased the risk of general and abdominal obesity (32). Our data reinforces this conclusion and provides the new insight that intra-meal water drinking is associated with an increased risk of CMM in women.

It is worth noting that prior research has concentrated on specific dietary behaviors, with little evidence of the combined impact of various high-risk dietary behaviors and CMM. Considering that the individual impacts of each high-risk dietary behavior may be too minor to detect, investigating the relationship between multiple dietary behaviors and CMM may reveal new insights into CMM management. Our data analysis indicates that engaging in two or more concurrent high-risk dietary behaviors is related to a greater likelihood of CMM occurrence than having either no high-risk dietary behaviors or just one, which applied to both sexes. Moreover, we predicted that specific combinations of high-risk dietary behaviors might affect the likelihood of developing CMM. We examined serval potential patterns of high-risk dietary behaviors using LCA in CMM patients stratified by gender. Our results suggest that while frequent night snacking has no effect on the risk of CMM in men and women, participants with frequent night snacking may commonly engage in other high-risk dietary behaviors. The results support that participants with two or more high-risk dietary behaviors had a higher risk of developing CMM. Identifying individuals with high-risk dietary behaviors, such as frequent night snacking, and intervening with a focus on modulating their dietary behavior patterns may thus benefit the achievement of a healthy cardiometabolic status (31). Future research should focus on the underlying connectivity of several high-risk dietary behavior clusters to help develop individualized dietary behavior management strategies for CMM patients.

In addition, we examined the taste preferences of individuals to investigate their relationship with CMM. Chongqing locals are characterized by an overall preference for spicy food due to their region's topography and climatic factors, which is consistent with our findings. According to our data analysis, Chongqing inhabitants' top four dietary taste preferences were spicy, salty, sweet, and greasy, in that order. A higher frequency and intensity of spicy food intake (47) as well as an oil- and salt-rich diet (48) have been reported to be associated with unfavorable cardiovascular disease risk profiles in middle-aged and older adults, which is generally consistent with our findings. However, we investigated these relationships further in gender-segregated subgroups. In particular, taste preferences for smoky, hot, and spicy foods in men were more highly associated with CMM risk. This difference could be attributed to the higher proportion of men reporting taste preferences among the participants in this study. More research is needed to establish the gender variations in taste preferences associations with CMM and to elucidate the underlying mechanisms.

This study had several limitations that should be considered when interpreting the results. First, the temporal bias in cross-sectional studies prevents cause-and-effect linkages from being established, and future longitudinal research is needed to overcome this. Second, CMM was measured by self-reported chronic conditions, and hence may have been under- or over-reported in this study. Furthermore, memory bias may affect self-reported lifestyle data, and the dichotomous classification of lifestyles may lead to unintentional misclassification. For example, we did not consider the differential effects between under-intake and excessive food intake on CMM. Third, the factors in this study could not be all-inclusive because they are based on real-world data. All pertinent factors could not be considered, including participant sleep quality, BMI, and more specific dietary behavior data. Furthermore, due to participants' concerns about information privacy and study time constraints, socio-demographic information; such as economic income and educational attainment; were not collected in this study. Finally, we urge careful consideration of the results because the study's data were gathered from a single hospital health management center, which may not be representative of the wider population or other subpopulations. Further research is needed to confirm our findings, explicitly expanding the study coverage to include more comprehensive lifestyle factors and detailed information on dietary behavior. Our findings may help develop CMM management strategies and allocate healthcare services. The findings could be helpful for other low- and middle-income countries characterized by similar topography and climate.

## Conclusion

The accumulation of poor lifestyle factors increases the risk of CMM in middle-aged and older patients in Chongqing, Southwest China. Although the associations differed by gender, unhealthy dietary behaviors (such as over-eating and intra-meal water drinking) and taste preferences (such as smoky, hot, and spicy) were linked to an increased risk of CMM. The necessity for a change in the health care system to assess and modulate multiple behaviors for multiple diseases is further explained by the several common high-risk lifestyle patterns and dietary behavior patterns. Our results show that identifying people with certain high-risk lifestyle factors, such as high-risk physical activity, and managing them with a focus on their lifestyle patterns may assist patients with CMM to achieve better health outcomes. Further research is required to elucidate potential underlying mechanisms and establish causation between these lifestyle factors and CMM, particularly prospective and interventional investigations.

## Data availability statement

The raw data supporting the conclusions of this article will be made available by the authors, without undue reservation.

## Ethics statement

The studies involving human participants were reviewed and approved by the Ethics Committee of the First Affiliated Hospital of Chongqing Medical University (2020426). The patients/participants provided their written informed consent to participate in this study.

## Author contributions

YZ: conceptualization, methodology, and writing—original draft. ZZ: methodology, data analytics, and visualization. TW: writing—review and editing. RS: data collection and curation. KZ, HH, and HZ: data curation and writing—original draft. WL: conceptualization, funding acquisition, and project administration. All authors contributed to the article and approved the final version.
