# Supplementary material for: Association between composite lifestyle factors and cardiometabolic multimorbidity in Chongqing, China: A cross-sectional exploratory study in people over 45 years and older
Source: Front Public Health. 2023 Feb 1;11:1118628. doi: 10.3389/fpubh.2023.1118628 (PMC9929179; doi:10.3389/fpubh.2023.1118628)
Supplement: Supplementary file 1 [file Table_1.DOCX]

Supplementary Material

# Supplementary Tables

| **Supplementary Table 1 Fitting indexes for the latent class models (high-risk lifestyle, male).** | | | | | | | | |
| --- | --- | --- | --- | --- | --- | --- | --- | --- |
| Model | K | LL | AIC | BIC | ABIC | Entropy | LMR | BLRT |
| 1-class model | 5 | -3344.208 | 6698.416 | **6723.228** | 6707.347 | - | - | - |
| 2-class model | 11 | -3330.987 | 6683.975 | 6738.559 | **6703.622** | **0.906** | <0.01 | <0.01 |
| 3-class model | 17 | **-3322.25** | **6678.500** | 6762.858 | 6708.863 | 0.491 | 0.02 | 0.03 |

Note: K refers to Number of Free Parameters, LL refers to Log-likelihood, AIC refers to Akaike Information Criterion; BIC refers to Bayesian Information Criterion; ABIC refers to adjusted Bayesian Information Criterion; LMR refers to Lo-Mendell-Rubin adjusted likelihood ratio test; BLRT refers to bootstrapped likelihood ratio test.

| **Supplementary Table 2 Fitting indexes for the latent class models (high-risk lifestyle, female).** | | | | | | | | |
| --- | --- | --- | --- | --- | --- | --- | --- | --- |
| Model | K | LL | AIC | BIC | ABIC | Entropy | LMR | BLRT |
| 1-class model | 5 | -1196.954 | **2403.908** | **2426.04** | **2410.166** | - | - | - |
| 2-class model | 11 | -1191.686 | 2405.372 | 2454.063 | 2419.14 | 0.757 | 0.6692 | 0.2083 |
| 3-class model | 17 | **-1187.839** | 2409.678 | 2484.928 | 2430.956 | 0.999 | 0.1929 | 0.6667 |

Note: K refers to Number of Free Parameters, LL refers to Log-likelihood, AIC refers to Akaike Information Criterion; BIC refers to Bayesian Information Criterion; ABIC refers to adjusted Bayesian Information Criterion; LMR refers to Lo-Mendell-Rubin adjusted likelihood ratio test; BLRT refers to bootstrapped likelihood ratio test.

| **Supplementary Table 3 Fitting indexes for the latent class models (high-risk dietary behavior, male).** | | | | | | | | |
| --- | --- | --- | --- | --- | --- | --- | --- | --- |
| Model | K | LL | AIC | BIC | ABIC | Entropy | LMR | BLRT |
| 1-class model | 6 | -3064.618 | 6141.237 | 6171.01 | 6151.953 | - | - | - |
| 2-class model | 13 | -2995.516 | 6017.032 | **6081.541** | 6040.251 | 0.499 | <0.01 | <0.01 |
| 3-class model | 20 | -2981.6 | **6003.2** | 6102.445 | **6038.922** | **0.767** | <0.01 | <0.01 |
| 4-class model | 27 | **-2972.259** | 5998.518 | 6132.499 | 6046.743 | 0.638 | 0.523 | 0.05 |

Note: K refers to Number of Free Parameters, LL refers to Log-likelihood, AIC refers to Akaike Information Criterion; BIC refers to Bayesian Information Criterion; ABIC refers to adjusted Bayesian Information Criterion; LMR refers to Lo-Mendell-Rubin adjusted likelihood ratio test; BLRT refers to bootstrapped likelihood ratio test.

| **Supplementary Table 4 Fitting indexes for the latent class models (high-risk dietary behavior, female).** | | | | | | | | |
| --- | --- | --- | --- | --- | --- | --- | --- | --- |
| Model | K | LL | AIC | BIC | ABIC | Entropy | LMR | BLRT |
| 1-class model | 6 | -1611.986 | 3235.973 | 3262.532 | 3243.483 | - | - | - |
| 2-class model | 13 | -1567.798 | 3161.596 | **3219.14** | **3177.867** | **0.717** | <0.01 | <0.01 |
| 3-class model | 20 | **-1558.347** | **3156.693** | 3245.223 | 3181.727 | 0.565 | 0.7813 | 0.0606 |

Note: K refers to Number of Free Parameters, LL refers to Log-likelihood, AIC refers to Akaike Information Criterion; BIC refers to Bayesian Information Criterion; ABIC refers to adjusted Bayesian Information Criterion; LMR refers to Lo-Mendell-Rubin adjusted likelihood ratio test; BLRT refers to bootstrapped likelihood ratio test.

**Supplementary Table 5 Logistic regression of the independent effects of each food group on cardiometabolic multimorbidity.**

|  | **Male** | | | | **Female** | |
| --- | --- | --- | --- | --- | --- | --- |
|  | **OR** | | | **95% CI** | **OR** | **95% CI** |
| Fresh vegetables (ref. Good) |  | | |  |  |  |
| Poor | 1.14 | | | (0.97-1.34) | 1.16 | (0.95-1.41) |
| Fresh fruits (ref. Good) |  | | |  |  |  |
| Poor | 1.07 | | | (0.88-1.31) | 1.18 | (0.95-1.45) |
| Rice and flour staples (ref. Good) |  | | |  |  |  |
| Poor | 1.14 | | | (0.99-1.32) | 0.95 | (0.75-1.19) |
| Eggs (ref. Good) |  | | |  |  |  |
| Poor | 0.95 | | | (0.82-1.11) | 1.33 | (1.09-1.64) ** |
| Soybeans (ref. Good) |  | | |  |  |  |
| Poor | 0.70 | | | (0.52-0.94) * | 1.42 | (0.88-2.29) |
| Milk and milk-based products (ref. Good) | |  | |  |  |  |
| Poor | 0.91 | | | (0.54-1.53) | 0.98 | (0.51-1.88) |
| Meat and meat-based products (ref. Good) | | |  |  |  |  |
| Poor | 1.14 | | | (0.99-1.30) | 1.21 | (1.02-1.44) * |
| Fish and aquatic products (ref. Good) |  | | |  |  |  |
| Poor | 0.99 | | | (0.86-1.14) | 1.09 | (0.90-1.31) |
| Water consumption (ref. Good) |  | | |  |  |  |
| Poor | 1.03 | | | (0.88-1.19) | 1.36 | (1.09-1.71) ** |

**Note: Adjustment with age group and family history of cardiometabolic multimorbidity, *:P<0.05, **:P<0.01.**

**Supplementary Table 6 Characteristics of dietary intake, dietary behavior and taste preference of the participants (N=14,968).**

|  |  | **Total** | **Male(n=7,427)** | **Female(n=7,541)** | **P-value** |
| --- | --- | --- | --- | --- | --- |
| **Dietary intake** | |  |  |  |  |
| Rice and flour staples | ≤250g | 11077 | 4866(65.5%) | 6211(82.4%) | <0.001 |
|  | 250g~≤400g | 3530 | 2306(31.1%) | 1224(16.2%) |  |
|  | >400g | 361 | 255(3.4%) | 106(1.4%) |  |
| Meat and meat-based products | ≤40g | 8619 | 4098(55.2%) | 4521(60.0%) | <0.001 |
|  | 40g~≤75g | 5178 | 2646(35.6%) | 2532(33.6%) |  |
|  | >75g | 1171 | 683(9.2%) | 488(6.4%) |  |
| Fish and aquatic products | ≤40g | 3480 | 1346(18.1%) | 2134(28.3%) | <0.001 |
|  | 40g~≤75g | 8109 | 3951(53.2%) | 4158(55.1%) |  |
|  | >75g | 3379 | 2130(28.7%) | 1249(16.6%) |  |
| Eggs | ≤40g | 10783 | 5394(72.6%) | 5389(71.5%) | 0.129 |
|  | 40g~≤50g | 3951 | 1910(25.7%) | 2041(27.0%) |  |
|  | >50g | 234 | 123(1.7%) | 111(1.5%) |  |
| Milk and milk-based products | ≤300ml | 14600 | 7260(97.8%) | 7340(97.3%) | 0.095 |
|  | 300~≤500ml | 246 | 118(1.5%) | 128(1.7%) |  |
|  | >500ml | 122 | 49(0.7%) | 73(1.0%) |  |
| Soybeans | ≤25g | 14190 | 7037(94.7%) | 7153(94.9%) | 0.924 |
|  | 25~≤30g | 661 | 330(4.5%) | 331(4.3%) |  |
|  | >30g | 117 | 60(0.8%) | 57(0.8%) |  |
| Fresh vegetables | ≤300g | 10032 | 5127(69.0%) | 4905(65.0%) | <0.001 |
|  | 300~≤500g | 3810 | 1799(24.3%) | 2011(26.7%) |  |
|  | >500g | 1126 | 501(6.7%) | 625(8.3%) |  |
| Fresh fruits | ≤200g | 11616 | 6200(83.5%) | 5416(71.8%) | <0.001 |
|  | 200~≤350g | 2758 | 1038(14.0%) | 1720(22.8%) |  |
|  | >350g | 594 | 189(2.5%) | 405(5.4%) |  |
| Water consumption | ≤1500ml | 10501 | 4916(66.2%) | 5585(74.1%) | <0.001 |
|  | 1500~≤1700ml | 3491 | 1908(25.7%) | 1583(21.0%) |  |
|  | >1700ml | 976 | 603(8.1%) | 373(4.9%) |  |
| **Dietary behavior** |  |  |  |  |  |
| Breakfast irregularity | 7 days/week | 267 | 137(1.8%) | 130(1.7%) | <0.001 |
|  | 3-6 days/week | 286 | 168(2.3%) | 118(1.6%) |  |
|  | 1~2days/week | 2562 | 1470(19.8%) | 1092(14.5%) |  |
|  | Never | 11853 | 5652(76.1%) | 6201(82.2%) |  |
| Frequent night snacking | Never | 9037 | 4178(56.3%) | 4859(64.4%) | <0.001 |
|  | 1~2days/week | 1759 | 1061(14.3%) | 698(9.3%) |  |
|  | 3-6 days/week | 943 | 554(7.5%) | 389(5.2%) |  |
|  | 7 days/week | 3229 | 1634(22.0%) | 1595(21.2%) |  |
| Intra-meal water drinking | No | 14010 | 6944(93.5%) | 7066(93.7%) | 0.609 |
|  | Yes | 958 | 483(6.5%) | 475(6.3%) |  |
| Eating excessively fast | No | 11179 | 5078(68.4%) | 6101(80.9%) | <0.001 |
|  | Yes | 3789 | 2349(31.6%) | 1440(19.1%) |  |
| Over-eating | No | 13411 | 6558(88.3%) | 6853(90.9%) | <0.001 |
|  | Yes | 1557 | 869(11.7%) | 688(9.1%) |  |
| Eating excessively late for dinner | No | 13635 | 6746(90.8%) | 6889(91.4%) | 0.261 |
|  | Yes | 1333 | 681(9.2%) | 652(8.6%) |  |
| **Taste preference** |  |  |  |  |  |
| Salty | No | 13676 | 6636(89.3%) | 7040(93.4%) | <0.001 |
|  | Yes | 1292 | 791(10.7%) | 501(6.6%) |  |
| Sweet | No | 13682 | 6858(92.3%) | 6824(90.5%) | <0.001 |
|  | Yes | 1286 | 569(7.7%) | 717(9.5%) |  |
| Cold | No | 14615 | 7242(97.5%) | 7373(97.8%) | 0.289 |
|  | Yes | 353 | 185(2.5%) | 168(2.2%) |  |
| Hot | No | 14072 | 6890(92.8%) | 7182(95.2%) | <0.001 |
|  | Yes | 896 | 537(7.2%) | 359(4.8%) |  |
| Greasy | No | 13922 | 6708(90.3%) | 7214(95.7%) | <0.001 |
|  | Yes | 1046 | 719(9.7%) | 327(4.3%) |  |
| Smokey | No | 14328 | 7055(95.0%) | 7273(96.4%) | <0.001 |
|  | Yes | 640 | 372(5.0%) | 268(3.6%) |  |
| Spicy | No | 12070 | 5778(77.8%) | 6292(83.4%) | <0.001 |
|  | Yes | 2898 | 1649(22.2%) | 1249(16.6%) |  |
